# Plakophilin-1, a Novel Wnt Signaling Regulator, Is Critical for Tooth Development and Ameloblast Differentiation

**DOI:** 10.1371/journal.pone.0152206

**Published:** 2016-03-24

**Authors:** Kanako Miyazaki, Keigo Yoshizaki, Chieko Arai, Aya Yamada, Kan Saito, Masaki Ishikawa, Han Xue, Keita Funada, Naoto Haruyama, Yoshihiko Yamada, Satoshi Fukumoto, Ichiro Takahashi

**Affiliations:** 1 Section of Orthodontics and Dentofacial Orthopedics, Division of Oral Health, Growth and Development, Faculty of Dental Science, Kyushu University, Fukuoka, Japan; 2 Division of Pediatric Dentistry, Department of Oral Health and Development Sciences, Tohoku University Graduate School of Dentistry, Sendai, Japan; 3 Operative Dentistry, Department of Restorative Dentistry, Tohoku University Graduate School of Dentistry, Sendai, Japan; 4 Laboratory of Cell and Developmental Biology, National Institute of Dental and Craniofacial Research, National Institutes of Health, Bethesda, Maryland, United States of America; University of Texas Medical Branch, UNITED STATES

## Abstract

Tooth morphogenesis is initiated by reciprocal interactions between the ectoderm and neural crest-derived mesenchyme, and the Wnt signaling pathway is involved in this process. We found that Plakophilin (PKP)1, which is associated with diseases such as ectodermal dysplasia/skin fragility syndrome, was highly expressed in teeth and skin, and was upregulated during tooth development. We hypothesized that PKP1 regulates Wnt signaling via its armadillo repeat domain in a manner similar to β-catenin. To determine its role in tooth development, we performed *Pkp1* knockdown experiments using ex vivo organ cultures and cell cultures. Loss of *Pkp1* reduced the size of tooth germs and inhibited dental epithelial cell proliferation, which was stimulated by Wnt3a. Furthermore, transfected PKP1-emerald green fluorescent protein was translocated from the plasma membrane to the nucleus upon stimulation with Wnt3a and LiCl, which required the PKP1 N terminus (amino acids 161 to 270). Localization of PKP1, which is known as an adhesion-related desmosome component, shifted to the plasma membrane during ameloblast differentiation. In addition, *Pkp1* knockdown disrupted the localization of Zona occludens 1 in tight junctions and inhibited ameloblast differentiation; the two proteins were shown to directly interact by immunoprecipitation. These results implicate the participation of PKP1 in early tooth morphogenesis as an effector of canonical Wnt signaling that controls ameloblast differentiation via regulation of the cell adhesion complex.

## Introduction

Clarification of the mechanism of tooth morphogenesis is critical for understanding epithelial-mesenchymal organization of tissues and for developing tissue regeneration strategies. In mice, tooth development begins on embryonic day (E)11.5. The dental epithelium thickens to form the dental placode, which subsequently invaginates into the mesenchyme, forming the tooth bud at E13.5. One day later, the tooth bud progresses to the cap stage, with enamel knots forming in the dental epithelium, leading to tooth cusps. At the bell stage (E16.5), the dental epithelium develops into mesenchyme and forms the tooth crown. In the ameloblast differentiation stage [postnatal day (P)1-7], the inner dental epithelial cell layer differentiates into ameloblasts, which is accompanied by a change in cell shape to rectangular, polarization of the nucleus to the basal side, and secretion of enamel matrices. During this process, interactions between cells are essential for the development of all organs, including teeth. Molecules such as Occludin, Claudin, Zona occludens (ZO)-1, and Connexin 43 are expressed and accumulate at cell-cell junctions [1, 2]. The roles of cell adhesion molecules in tooth development have been studied in regard to their roles in the determination of tooth shape and size, and the function of enamel-producing ameloblasts [3, 4]. Although cell adhesion is critical for ameloblast differentiation and stabilization, the underlying molecular mechanisms remain unclear.

Wnt/β-catenin signaling is dynamically activated during tooth development and plays various roles in this process [5–8]. The Wnt signaling pathway is activated by the binding of Wnt protein to the Frizzled receptor; in the canonical pathway, this leads to the accumulation of β-catenin in the cytoplasm and its subsequent translocation to the nucleus, where it activates transcription in conjunction with T cell factor (TCF)/Lymphoid enhancer-binding factor (LEF) family members. Thus, the activation of β-catenin regulates organ development. Furthermore, expression of a mutated and constitutively active form of β-catenin in the epithelium causes tooth formation to fail, leading to formation of ectopic teeth [5]. Conversely, expression of an inactive form of β-catenin in mice results in the arrest of tooth formation at the bud stage [9]. Members of the armadillo protein family, including β-catenin and Plakoglobin, have a variety of functions in cytoskeleton–cell membrane interactions [10, 11]. In addition, they act as linker molecules at adherens junctions, desmosomes, and hemidesmosomes at the plasma membrane, and play important roles in signal transduction and modulation of cell behavior during tissue development [12, 13].

In the present study, we found that Plakophilin (PKP)1 was highly expressed in the teeth of E14 mouse embryos, as shown by microarray analysis. PKP1 is a member of the armadillo protein family and contains an armadillo repeat domain, the same as β-catenin, suggesting that it is a candidate effector protein for Wnt signaling. Mutations in the *Pkp1* gene result in ectodermal dysplasia/skin fragility syndrome, which affect skin, hair, and nails [14–17]. Although dental abnormalities have been reported in a few cases, the precise role of PKP1 in tooth development has not been reported. Here, we present findings showing that PKP1 acts as a Wnt signal transducer and translocates to the nucleus upon Wnt stimulation during early tooth development. We also found that it plays a critical role in ameloblast differentiation via interaction with cell adhesion molecules.

## Materials and Methods

### Microarray analysis

Total RNA was isolated from the first molar of the mandible and whole embryos at E14 with TRIzol reagent (Life Technologies, Carlsbad, CA, USA), then purified using an RNeasy Mini kit (Qiagen, Valencia, CA, USA) according to the manufacturer’s protocol. RNA quality was verified using an Experion automated electrophoresis system (Bio-Rad, Hercules, CA, USA), and found to be RQI = 10.0 and 9.9 for tooth and whole embryo RNA, respectively. Labeling and array hybridization were performed according to standard protocols at the Research Support Center of the Research Center for Human Disease Modeling of Kyushu University. Gene expression profiles were analyzed using a chip-based gene array (MouseWG-6 v2; Illumina, Santa Clara, CA, USA). Data were normalized using Genome Studio (Illumina). Gene expression analysis was carried out using Subio Platform v1.18 (Subio, Amami, Japan).

### Tissue preparation and histological analysis

All animal experiments were approved by the ethics committee of Kyushu University Animal Experiment Center (protocol no. A26-208-0). The pregnant mice were euthanized by anesthesia and then mouse embryos were dissected immediately. Embryo heads were fixed with 4% paraformaldehyde in phosphate-buffered saline (PBS) for 16 h at 4°C. Samples were incubated in gradient sucrose solutions in PBS for 12–24 h, then embedded in O.C.T. compound (Sakura Finetek, Tokyo, Japan). The heads were sectioned at 10 μm on a cryostat (CM 1800; Leica, Wetzlar, Germany). For immunolabeling, sections were fixed with 4% paraformaldehyde for 5 min, then treated with Liberate Antibody-binding Solution (Polysciences, Warrington, PA, USA) for 15 min at 37°C for antigen retrieval. Primary antibodies against PKP1 (1:500; Abcam, Cambridge, MA, USA), ZO-1 (1:500; Cell Signaling Technology, Danvers, MA, USA), Ameloblastin (AMBN, 1:100; Santa Cruz Biotechnology, Santa Cruz, CA, USA), and β-catenin (1:500; Sigma Aldrich, Saint Louis, MO, USA) were applied for 1 h at room temperature (RT). For immunolabeling of cells, cervical-loop derived dental epithelium (CLDE) cells were plated in the wells of a chambered slide (Thermo Fisher Scientific, Waltham, MA, USA), then fixed with 4% paraformaldehyde for 5 min and permeabilized with 0.05% Triton X-100 in PBS for 10 min at RT. Primary antibodies against PKP1 (1:500), Keratin 14 (1:1000; Covance, Princeton, NJ, USA), Desmoplakin 1+2 (1:200; Abcam), and E-cadherin and ZO-1 (both at 1:500; both from Cell Signaling Technology) were applied for 1 h at RT. Cells and tissue sections were then incubated with species-specific secondary antibodies conjugated with Alexa 488 or Alexa 594 fluorescent dye (Life Technologies) for 1 h at RT. To visualize nuclei, cells and tissue sections were mounted with Vectashield mounting medium containing DAPI (Vector Laboratories, Burlingame, CA, USA). Images were captured with a C2 confocal microscope (Nikon, Tokyo, Japan) and analyzed using NIS-Elements AR software v4.00 (Nikon).

### Cell culture and transfection

CLDE cells were obtained as previously reported [18] and maintained in Ca^2+^-free keratinocyte serum-free medium (K-SFM, 37010–022; Gibco/Life Technologies) supplemented with epidermal growth factor, bovine pituitary extract, and 1% penicillin/streptomycin (Gibco/life Technologies) at 37°C in a humidified atmosphere of 5% CO_2_. For the differentiation assay, cells were cultured with 100 ng/ml Neurotrophin (NT)-4 or 1.5 mM Ca^2+^. For transfection of a PKP1 expression vector or short interfering (si)RNA against *Pkp1*, cells were seeded in 12-well plates at a density of 2 × 10^5^ cells/well in K-SFM, then transfected with the expression vector using Lipofectamine 3000 with Plus reagent (Life Technologies) according to the manufacturer’s protocol. Cells were transfected with siRNA against *Pkp1* (ON-TARGET Plus L-049579-01; Dharmacon, Lafayette, CO, USA) or control siRNA (D-001810-10; Thermo Fisher Scientific) using Lipofectamine 3000 without Plus reagent. To activate the Wnt signaling pathway, 5 ng/ml Wnt3a (R&D Systems, Minneapolis, MN, USA) or 40 mM LiCl [19] was added to cells in the presence or absence of Ca^2+^.

### Cell proliferation and bromodeoxyuridine (BrdU) incorporation

CLDE cells were cultured in 96-well plates at 0.8 × 10^4^ cells/well for 72 h, then transfected with control or *Pkp1* siRNA. Proliferation was determined using a Cell Counting Kit (CCK)-8 (Dojindo Laboratories, Kumamoto, Japan) according to the manufacturer’s protocol. Briefly, 10 μl of CCK-8 solution was added to each well and optical density was measured at 450 nm using an iMark microplate reader (Bio-Rad). Cell proliferation was also assayed by BrdU incorporation using a BrdU labeling kit (Roche Diagnostics, Indianapolis, IN, USA). Cells transfected with control or *Pkp1* siRNA were cultured for 48 h. BrdU was added to the plates for 30 min, then incorporated BrdU was detected after 3 washes in PBS according to the manufacturer’s protocol. BrdU-positive cells were counted and the incorporation ratio was calculated by visualizing the cells with a confocal microscope.

### Organ culture

Mandibular molar tooth germs were dissected from E13 embryos from imprinting control region mice, seeded on a cell culture insert (BD Falcon; BD Biosciences, Franklin Lakes, NJ, USA), and grown using an air-liquid interface culture technique in Dulbecco’s Modified Eagle’s Medium/F12 supplemented with 20% fetal bovine serum (Gibco/Life Technologies), 180 μg/ml ascorbic acid, 2 mM l-glutamine, and 50 U/ml penicillin/streptomycin at 37°C in a humidified atmosphere of 5% CO_2_ for 7 days, as previously described [20–22]. For siRNA-mediated knockdown, cells were transfected with control or *Pkp1* siRNA at a concentration of 500 nM. Briefly, tooth germs were seeded on a cell culture insert, then transfected with siRNA against *Pkp1* or control siRNA using Lipofectamine 3000 according to the manufacturer’s protocol. To examine the knock down efficiency, tooth germs cultured for 48 hours were collected and total RNA was isolated with TRIzol reagent. Relative *Pkp1* expression levels were measured by qRT-PCR, using a CFX Connect Real-Time PCR detection system (Bio-Rad).

### Luciferase assay

TCF/LEF activity was determined using a Cignal TCF/LEF Reporter Assay kit (luc) (Qiagen). CLDE cells were transfected with a TCF/LEF reporter plasmid, then cultured with Ca^2+^ for 24 h and stimulated with Wnt3a for 24 h, after which luciferase activity was determined using a Dual-Luciferase Reporter Assay System (Promega, Madison, WI, USA) with a luminometer (Berthold Technologies, Oak Ridge, TN, USA). The activity was normalized to that of Renilla luciferase, which served as the internal control.

### RNA isolation and quantitative real-time (qRT)-PCR analysis

Total RNA of cultured cells was isolated using an RNeasy Mini kit. Tissue samples were dissected at specific embryonic stages, then total RNA was isolated using TRIzol reagent and purified using a RNeasy Mini kit. cDNA was synthesized from 2 μg of total RNA using random hexamers (Life Technologies) and SuperScript III Reverse Transcriptase (Life Technologies). The specific forward and reverse primers used for qRT-PCR were as follows: *Pkp1*, 5'-caacactgcctcctcaccta-3' and 5'-cctatcgaaaccttgctgcc-3'; *Cttnb1*, 5’-tcatcattctggccagtggt-3’ and 5’-tctgtcagatgaagccccag-3’; and *Glyceraldehyde 3-phosphate dehydrogenase* (*Gapdh*; internal control), 5'-ggagcgagaccccactaacatc-3' and 5'-ctcgtggttcacacccatcac-3'. Primers for AMBN have been reported [23]. qRT-PCR was performed using iQ SYBR Green Supermix (Bio-Rad) with a CFX Connect Real-Time PCR detection system (Bio-Rad). Relative mRNA levels were compared to that of *Gapdh* using the ∆∆Ct method.

### Western blotting

Total protein from cells was extracted with CelLytic M (Sigma-Aldrich) supplemented with 1% protease inhibitor cocktail (Sigma-Aldrich) and 1 mM phenylmethylsulfonyl fluoride (Sigma-Aldrich). Protein concentration was determined using a Bicinchoninic Acid Protein Assay kit (Thermo Fisher Scientific), and 10 μg protein from each sample was resolved by 4–12% sodium dodecyl sulfate polyacrylamide gel electrophoresis (Life Technologies), transferred to a polyvinylidene difluoride membrane (Life Technologies), and incubated with antibodies against PKP1 (1:500), ZO-1 (1:500), E-cadherin (1:500), β-catenin (1:500; Cell Signaling Technology), AMBN (1:100; Santa Cruz Biotechnology), Sex-determining region-Y-box (Sox)2 (1:500; Abcam), and GAPDH (1:500; Cell Signaling Technology), followed by incubation with horseradish peroxidase-conjugated secondary antibodies. The membrane was developed with ECL Plus reagent (Thermo Fisher Scientific).

### Nuclear translocation assay

Cells were plated in Nunc Lab-Tek II 8-well chambered slides (Thermo Fisher Scientific) at 2 × 10^4^ cells/well, then transfected with emerald green fluorescent protein (Em-GFP)-tagged PKP1-FL, PKP1-N1, PKP1-N2, and β-catenin expression vectors using Lipofectamine 3000. For membrane localization of PKP1 and β-catenin, cells were stimulated with 1.5 mM Ca^2+^ for 48 h. For nuclear translocation, transfected cells were treated with 5 ng/ml Wnt3a or 0–80 mM LiCl, then fixed 24 h later with 4% paraformaldehyde and stained with DAPI. The cell numbers showing Em-GFP in nucleus were divided by the total Em-GFP positive cell numbers to calculate the nuclear translocation ratio.

### Construction of expression vector

PKP1 and β-catenin expression vectors were constructed using a Gateway cloning system (Life Technologies) according to the manufacturer’s protocol. Briefly, the coding sequences of mouse PKP1 and β-catenin without a stop codon were cloned into a pENTR/D-TOPO entry vector. cDNA was prepared from E14 tooth mRNA by reverse transcription PCR and the sequences were confirmed by DNA sequencing. The following forward and reverse primers were used: PKP1-FL, 5'-caccatgaaccactctccgctcaa-3' and 5'-gaaccgggaggtgaagttt-3'; PKP1-delN1, 5'-caccatgtactgtgacccaaggggcacact-3' and 5'-gaaccgggaggtgaagttt-3'; PKP1-delN2, 5'-caccatgccagcacacctgcttccaggatgaat-3' and 5'-gaaccgggaggtgaagttt-3'; and β-catenin, 5'-caccatggctactcaagctgacctgat-3' and 5'-caggtcagtatcaaacca-3'. Expression vectors were cloned via an LR recombination reaction between the entry clone and destination vectors (Vivid Colors pcDNA6.2/C-EmGFP-DEST and pcDNA-DEST40, respectively; Life Technologies), then tagged with Em-GFP and V5-His, respectively.

### Immunoprecipitation assay

Cells were seeded into 10-cm dishes at a density of 1 × 10^6^/dish and cultured for 1 week in the presence of Ca^2+^, then harvested for protein extraction. Immunoprecipitation was carried out using a Dynabeads Protein G kit (Life Technologies) according to the manufacturer’s protocol. Antibodies against PKP1 and ZO-1, as well as normal rabbit IgG (Cell Signaling Technology) were fused to protein G magnetic beads and incubated with samples for 1 h at 4°C. The complex was eluted and denatured using NuPAGE LDS sample buffer (Life Technologies) supplemented with 1% 2-mercaptoethanol. Samples were analyzed by western blotting.

### Statistical analysis

All experiments were repeated 5 times to confirm reproducibility. Statistical significance was determined using the two-tailed unpaired Student’s t test with Prism 6 (GraphPad Software, La Jolla, CA, USA). Differences with P values < 0.05 were considered to be statistically significant.

## Results

### PKP1 is highly expressed during tooth development

**Fig 1:**
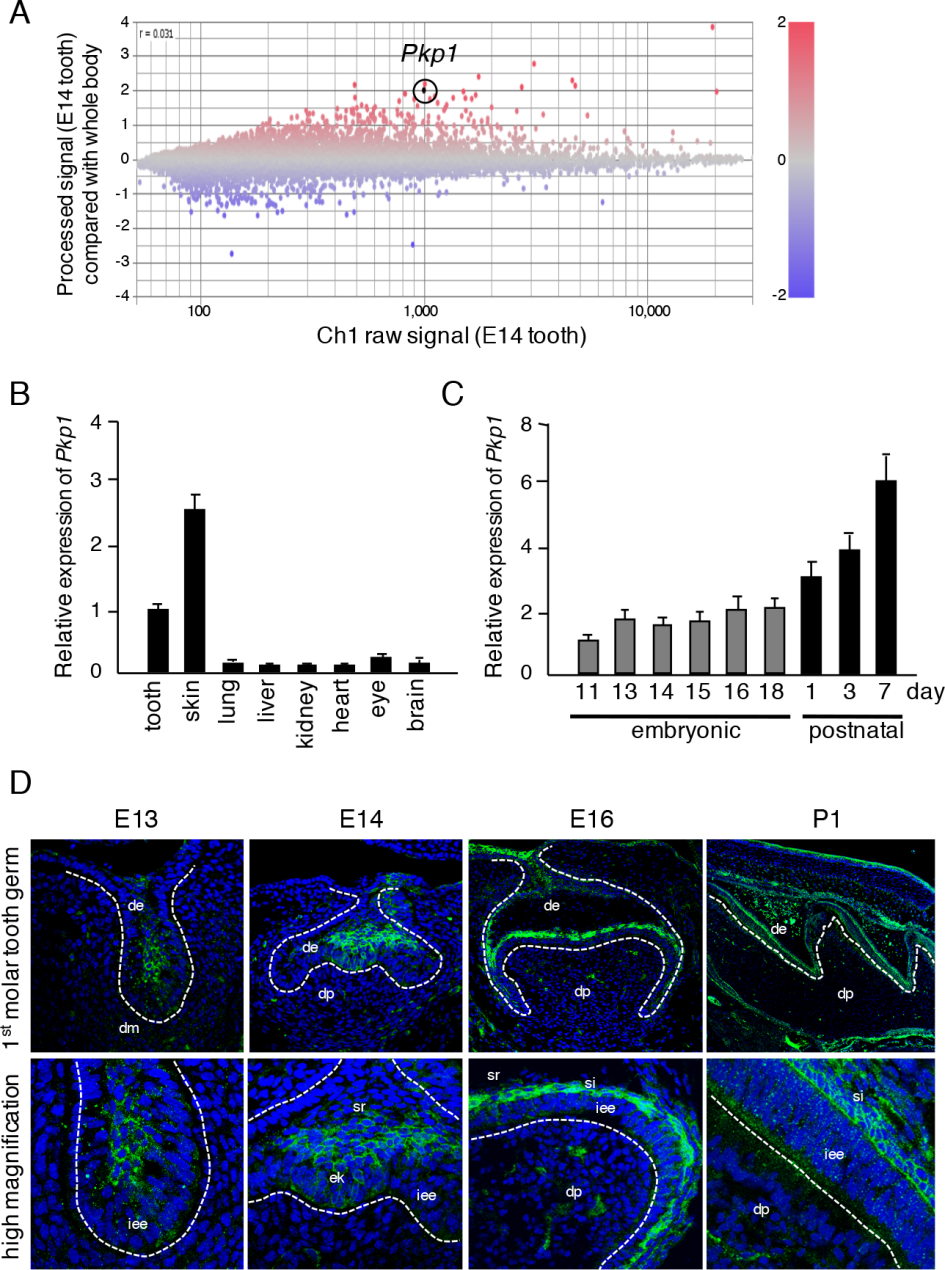
PKP1 expressed in developing tooth germs. A, Differentially expressed genes identified by microarray analysis of teeth as compared to whole embryos at E14. Highlighted plot indicates *Pkp1*. Red and blue plots represent up- and down-regulated genes, respectively. B, qRT-PCR analysis of *Pkp1* expression in teeth, skin, lungs, livers, kidneys, hearts, eyes, and brains of E14.5 embryos after normalization to *Gapdh* mRNA expression. C, qRT-PCR analysis of *Pkp1* expression in teeth obtained from E11 to P7 a normalization to *Gapdh* mRNA expression. D, PKP1 (green) expression in E13, E14, E16, and P1 mice, as detected by immunocytochemistry. Broken lines represent basement membrane of teeth. Enlarged images are shown below each panel. de, dental epithelium; dm, dental mesenchyme; iee, inner enamel epithelium; ek, enamel knot; sr, stellate reticulum; si, stratum intermedium; dp, dental papilla.

To identify tooth-specific genes at the morphogenesis stage, we performed microarray analysis to compare gene expressions in teeth with respect to the whole body at E14. A variety genes were found to be both up- and down-regulated in E14 teeth (Fig 1A). In the present study, we focused on *Pkp1*, which was shown to be highly expressed in teeth relative to the whole body. PKP1 has been implicated in ectodermal dysplasia/skin fragility syndrome, which is associated with tooth anomalies such as missing teeth and enamel hypoplasia. To confirm *Pkp1* expression during tooth development, we performed qRT-PCR using total RNA from teeth, skin, lungs, livers, kidneys, hearts, and eyes obtained at each stage of tooth developmental, i.e., E11, E13, E14, E15, E16, and E18, as well as on postnatal day (P)1, P3, and P7. *Pkp1* expression was high in teeth and skin (Fig 1B), and increased during the postnatal ameloblast differentiation stage (Fig 1C), implying that it plays an important role in tooth development and specifically in ameloblast differentiation. On the other hand, β-catenin was found to be ubiquitously expressed in tissues (S1A Fig). Furthermore, β-catenin expression during tooth germ development was at a high level in the embryonic stages and then diminished during the postnatal period (S1B Fig). These findings were confirmed by immunohistochemistry using lower first molars obtained on E13, E14, E16, and P1 (Fig 1D). PKP1 was mainly detected in epithelium, especially in E13 tooth bud. At E14, PKP1 was upregulated in stellate reticulum cells. However, at E16, its expression was mainly observed in the stratum intermedium and inner dental epithelial cells. At P1 during molar growth, ameloblast plasma membrane localization of PKP1 was detected, which was in contrast to the early tooth development stages (E13-E16) when PKP1 localization was cytosolic. On the other hand, β-catenin was highly expressed in stellate reticulum cells and mesenchymal tissues outside of the tooth germ, and found to be expressed in both dental epithelium and dental papilla on P1 (S1C Fig). These results indicate that PKP1 is highly expressed in dental epithelial cells, with its localization shifting from the cytosol to plasma membrane during tooth development.

### PKP1 regulates dental epithelium tooth germ size and cell proliferation

**Fig 2:**
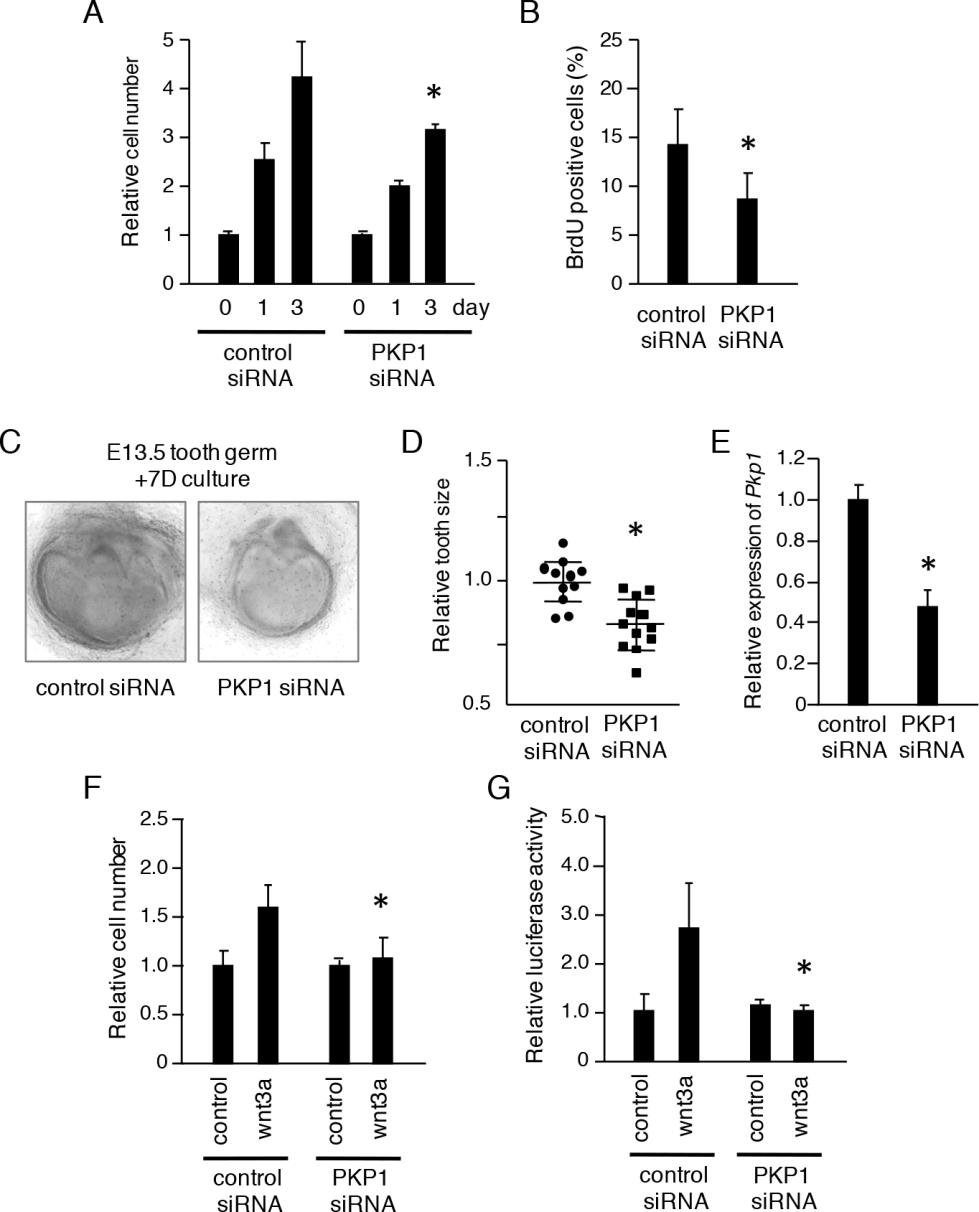
Pkp1 knockdown inhibits tooth germ growth and dental epithelial cell proliferation. A, CLDE cell proliferation 3 days after treatment with control or *Pkp1* siRNA, as determined by CCK-8 assay. B, BrdU incorporation by CLDE cells with or without *Pkp1* knockdown. The ratio was calculated as BrdU-positive cell number/DAPI-stained nuclei. C, Seven-day organ cultures of E13 tooth germs transfected with control or *Pkp1* siRNA. D, Relative tooth size plot (n = 12), with the average tooth germ size in the control siRNA group set at 1.0. E, qRT-PCR analysis of *Pkp1* expression in cultured tooth germ after normalization to *Gapdh* mRNA expression. F, CLDE cell proliferation in the presence of Wnt3a was evaluated using a CCK-8 assay after transfection with control or *Pkp1* siRNA. G, TCF/LEF promoter activity after stimulation with Wnt3a was examined using a luciferase assay after transfection with control or *Pkp1* siRNA. *P <0.01, Error bars represent the mean ± S.D.

PKP1 expression was first detected during the early stages of tooth development, suggesting that it is involved in tooth morphogenesis. We previously generated a CLDE dental epithelial cell line from the cervical loop of E15 mouse incisors, which contains dental epithelial stem cells [18]. To evaluate dental epithelial cell proliferation, we used CLDE cells in which *Pkp1* expression was knocked down, resulting in down-regulation of the transcript by 80% (data not shown). CLDE cells were transfected with control or *Pkp1* siRNA, and proliferation was evaluated with a CCK-8 assay. Loss of *Pkp1* decreased cell proliferation after 3 days of culture (Fig 2A) and reduced BrdU incorporation (Fig 2B), suggesting that *Pkp1* is required for epithelial cell proliferation. We also performed this analysis using an ex vivo organ culture system with lower molars dissected from E13 embryos. Tooth germ size was reduced following *Pkp1* knockdown as compared to control siRNA treatment (P <0.01) (Fig 2C and 2D). The knockdown efficiency of PKP1 siRNA for tooth germs was 50%, determined by qRT-PCR (Fig 2E). Wnt signaling, which is important for epithelial cell proliferation, induced proliferation of CLDE cells treated with Wnt3a (Fig 2F), an effect that was abolished by loss of *Pkp1* (Fig 2F), which also suppressed Wnt-induced TCF/LEF promoter activity (Fig 2G). These results suggest that PKP1 modulates tooth development by regulating dental epithelial cell proliferation via Wnt signaling.

### PKP1 is translocated to the cell membrane upon extracellular Ca^2+^ stimulation

**Fig 3:**
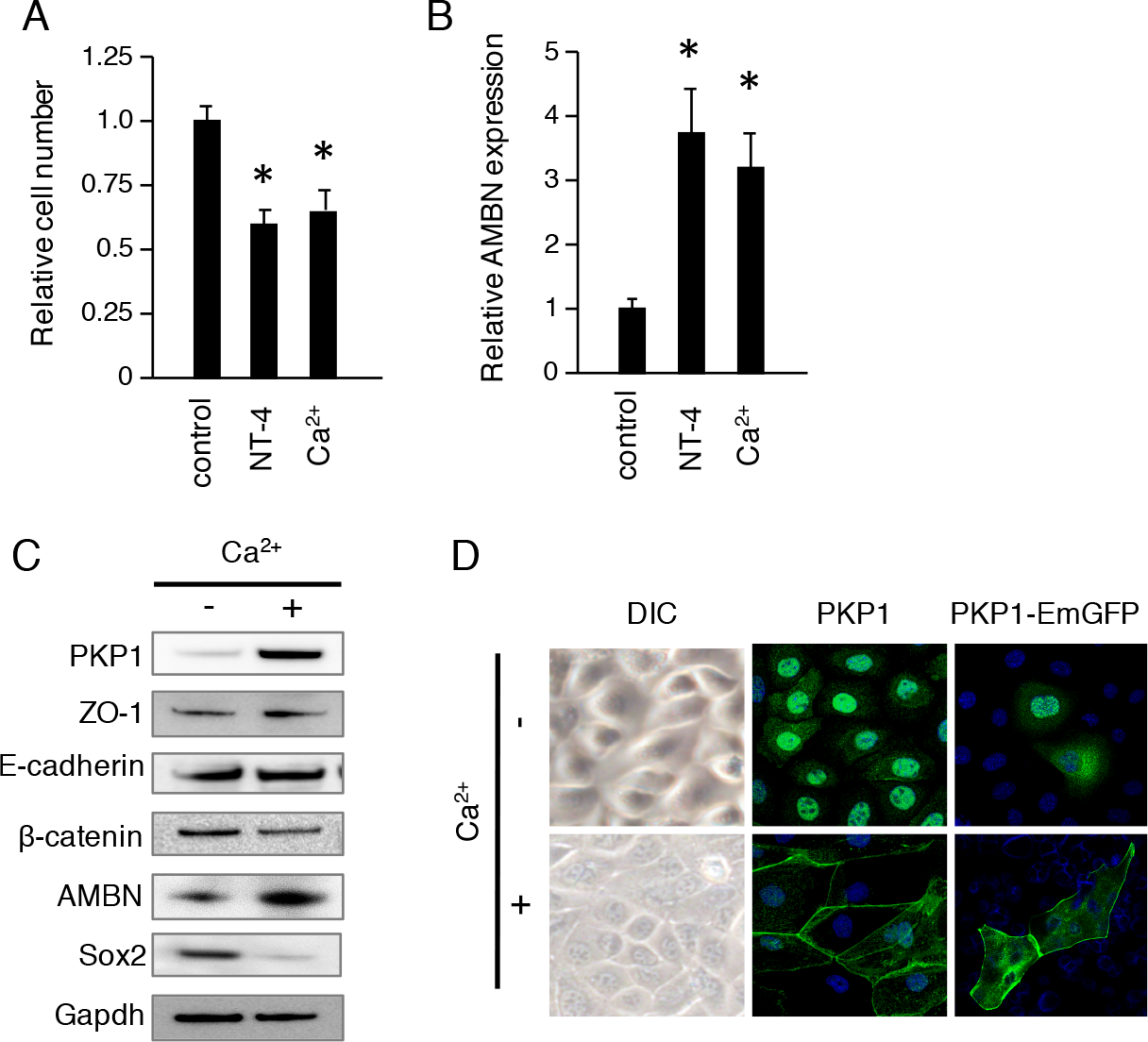
Extracellular Ca^2+^ induces PKP1 translocation from nucleus to plasma membrane. A, Cell proliferation was analyzed using a CCK-8 assay after stimulation with 100 ng/ml NT-4 or 1.5 mM Ca^2+^. B, AMBN expression was analyzed by qRT-PCR after stimulation with 100 ng/ml NT-4 or 1.5 mM Ca^2+^ (*P <0.01). Error bars represent the mean ± S.D. C, CLDE cells were cultured with 1.5 mM Ca^2+^ for 1 week, then the expressions of PKP1, ZO-1, E-cadherin, β-catenin, AMBN, and Sox2 were assessed by western blotting. GAPDH was used as a loading control. D. CLDE cells were cultured with or without Ca^2+^ for 48 h. Expressions of PKP1 (green, center panel) and transfected PKP1-EmGFP were detected by immunocytochemistry, and visualized by confocal microscopy. Nuclei were stained with DAPI (blue).

We previously showed that NT-4 induced ameloblast differentiation in dental epithelium [23, 24]. In the present study, NT-4 and extracellular Ca^2+^ inhibited proliferation (Fig 3A) and induced expression of the ameloblast differentiation marker AMBN in CLDE cells (Fig 3B). Extracellular Ca^2+^ is known to play a critical role in cell adhesion. To determine how it modulates dental epithelial cell differentiation, we analyzed protein expression in CLDE cells stimulated with extracellular Ca^2+^ by western blotting. AMBN expression was induced by Ca^2+^ treatment, while the dental epithelial stem cell marker Sox2 was down-regulated after 7 days of culture (Fig 3C). PKP1 was strongly induced by extracellular Ca^2+^ stimulation, which also altered CLDE cell shape into a paving stone arrangement and induced the translocation of endogenous PKP1 from the nucleus to the plasma membrane (Fig 3D). Furthermore, membrane localization of Pkp-1 and β-catenin induced by extracellular Ca^2+^ stimulation was diminished by Wnt3a stimulation (S2A and S2B Fig). These results indicate that extracellular Ca^2+^ stimulation increases PKP1 expression and alters its localization during the initial phase of ameloblast differentiation.

### Wnt activation induces the nuclear translocation of PKP1

**Fig 4:**
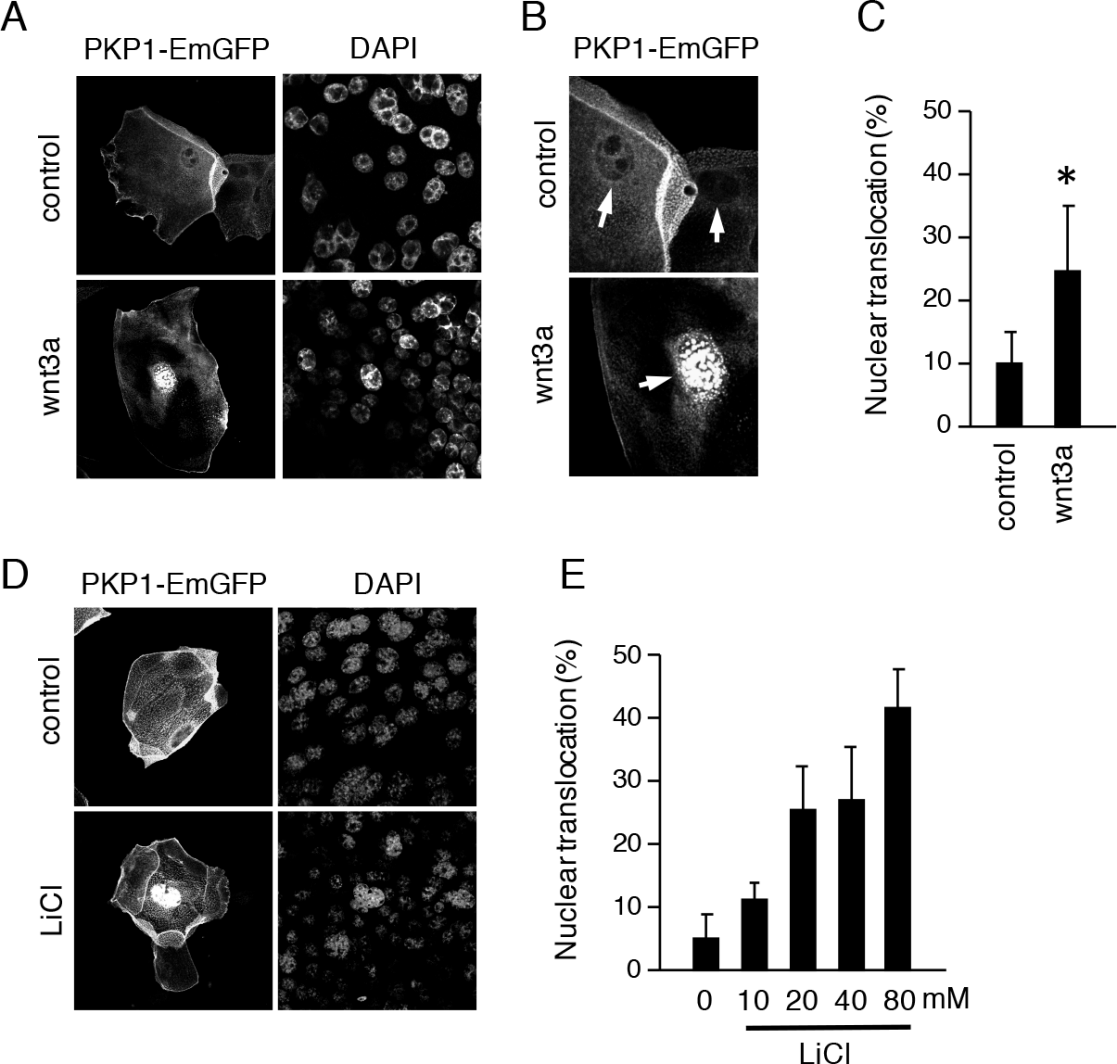
Wnt signaling induces translocation of PKP1 from membrane to nucleus. A, CLDE cells were transfected with PKP1-EmGFP, then treated with or without Wnt3a for 24 h. Nuclear translocation was detected by confocal microscopy. Nuclei were stained with DAPI. B, Enlarged image of PKP1-EmGFP nuclear translocation following stimulation with Wnt3a. C, Nuclear translocation ratio of CLDE cells treated with Wnt3a (*P <0.01). Error bars represent the mean ± S.D. D, CLDE cells were transfected with PKP1-EmGFP, then treated with 40 mM LiCl. Nuclear translocation was detected by confocal microscopy. E, Nuclear translocation ratios with different doses of LiCl in CLDE cells.

PKP1 is a member of the armadillo repeat domain protein family that includes β-catenin. PKP1 localizes in the cytosol during the early stages of tooth development (E13-14) (Fig 1D), suggesting that it is linked to Wnt signaling. We found that Wnt3a induced proliferation in CLDE cells (Fig 2F). To determine whether PKP1 translocates to the nucleus upon Wnt stimulation, CLDE cells were transfected with a PKP1-EmGFP expression vector and then cultured in the presence of Wnt3a. After 24 h, PKP1-EmGFP was translocated from the plasma membrane to the nucleus (Fig 4A–4C). The same result was observed at 24 h after application of LiCl (Fig 4D and 4E), which inhibits GSK3β and leads to activation of the Wnt signaling pathway. In addition, β-catenin-EmGFP was translocated from the plasma membrane to nucleus after stimulation with Wnt3a (S2C Fig), indicating that PKP1 is a downstream molecule in the canonical Wnt signaling pathway, the same as β-catenin.

### N terminus of PKP1 is required for LiCl-induced nuclear translocation

**Fig 5:**
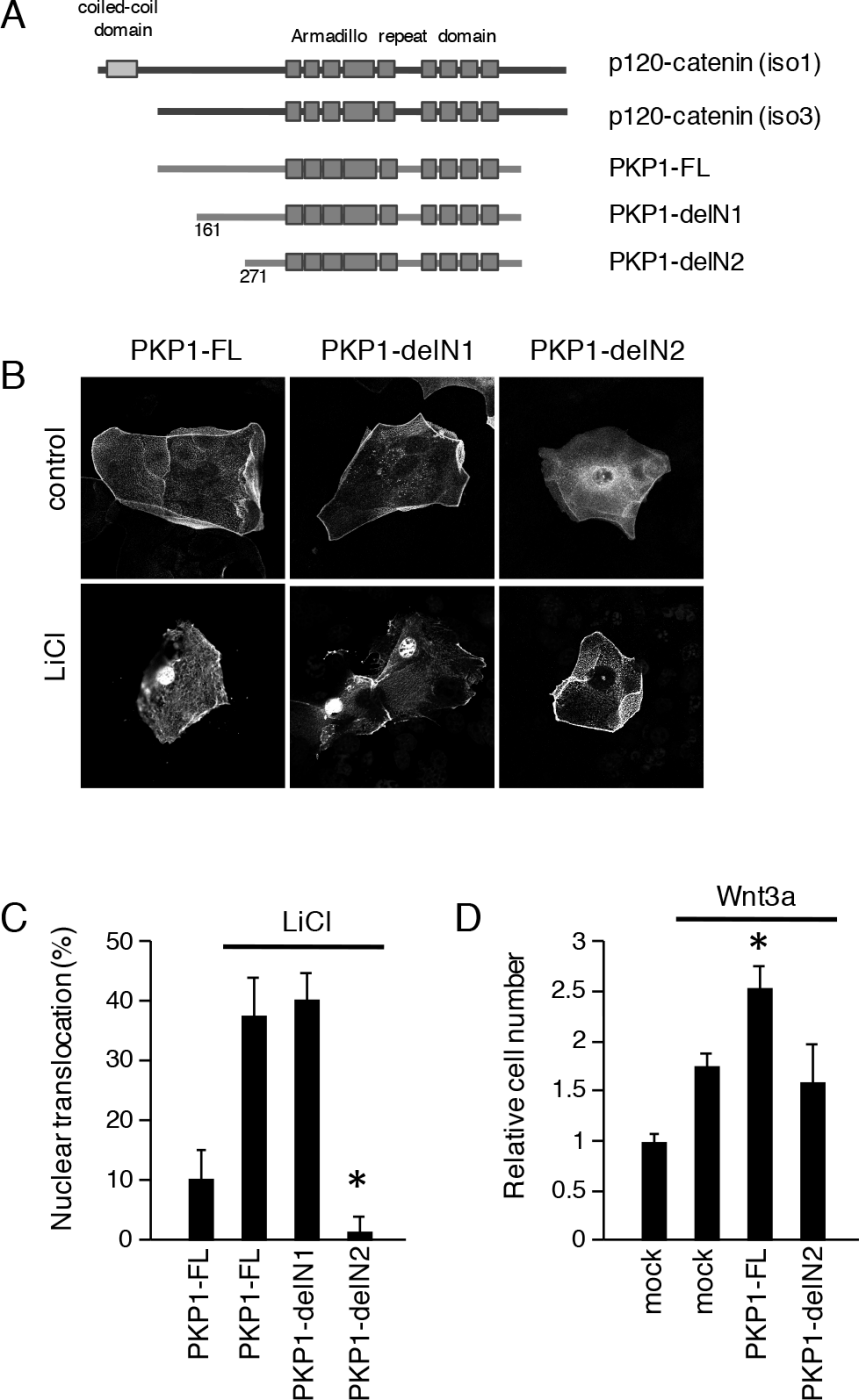
N terminus of PKP1 is required for nuclear translocation. A, Schematic representation of p120-catenin and PKP1-EmGFP constructs. PKP1-FL, full-length PKP1; PKP1-delN1, with deletion of N-terminal a.a. 1–160; PKP1-delN2, with deletion of N-terminal a.a. 1–270. All constructs contained the armadillo repeat domain. B, Nuclear translocation of PKP1-FL, -delN1, and -delN2 expressed in CLDE cells with or without LiCl treatment, as detected by confocal microscopy. C, Nuclear translocation ratios of CLDE cells transfected with PKP1-FL, -delN1, and -delN2 with or without LiCl treatment. D, Cell proliferation was determined after 72 h of culture by counting relative numbers of CLDE cells transfected with PKP1-FL and -delN2 with or without Wnt3a (*P <0.01). Error bars represent the mean ± S.D.

The PKP1 armadillo repeat domain is similar to that of p120-catenin, as it contains 9 repeats [25] (Fig 5A). Similar to β-catenin, PKP1 was shown to translocate to the nucleus of dental epithelial cells, implying that it contains a nuclear localization sequence. To test our speculation, we generated N-terminal-deleted PKP1 expression vectors that retained the armadillo repeat (Fig 5A) and examined nuclear localization of the expressed proteins following LiCl stimulation. As previously shown, PKP1-FL-EmGFP translocated to the nucleus in the presence of LiCl. Interestingly, nuclear localization was abolished for PKP1-delN2, lacking amino acids (a.a.) 1–270, but not for PKP1-delN1, lacking a.a. 1–160 (Fig 5B and 5C). These results suggest that an N-terminal region between a.a. 161 and 270 is required for nuclear localization of PKP1 and may regulate proliferation. Thus, we transfected CLDE cells with PKP1-FL and PKP1-N2 constructs tagged with V5-His, and assessed their proliferative capacity. Transfection of PKP1-FL increased proliferation as compared to mock-transfected control cells, while PKP1-N2 had no effect on proliferation after 72 h. Furthermore, transfection of PKP1-FL, but not PKP1-delN2, increased the proliferation of CLDE cells after stimulation with Wnt3a (Fig 5D). These results suggest that a.a. 161–270 are critical for nuclear translocation and regulation of cell proliferation via stimulation of Wnt signaling.

### PKP1 co-localizes with ZO-1 in differentiating ameloblasts

**Fig 6:**
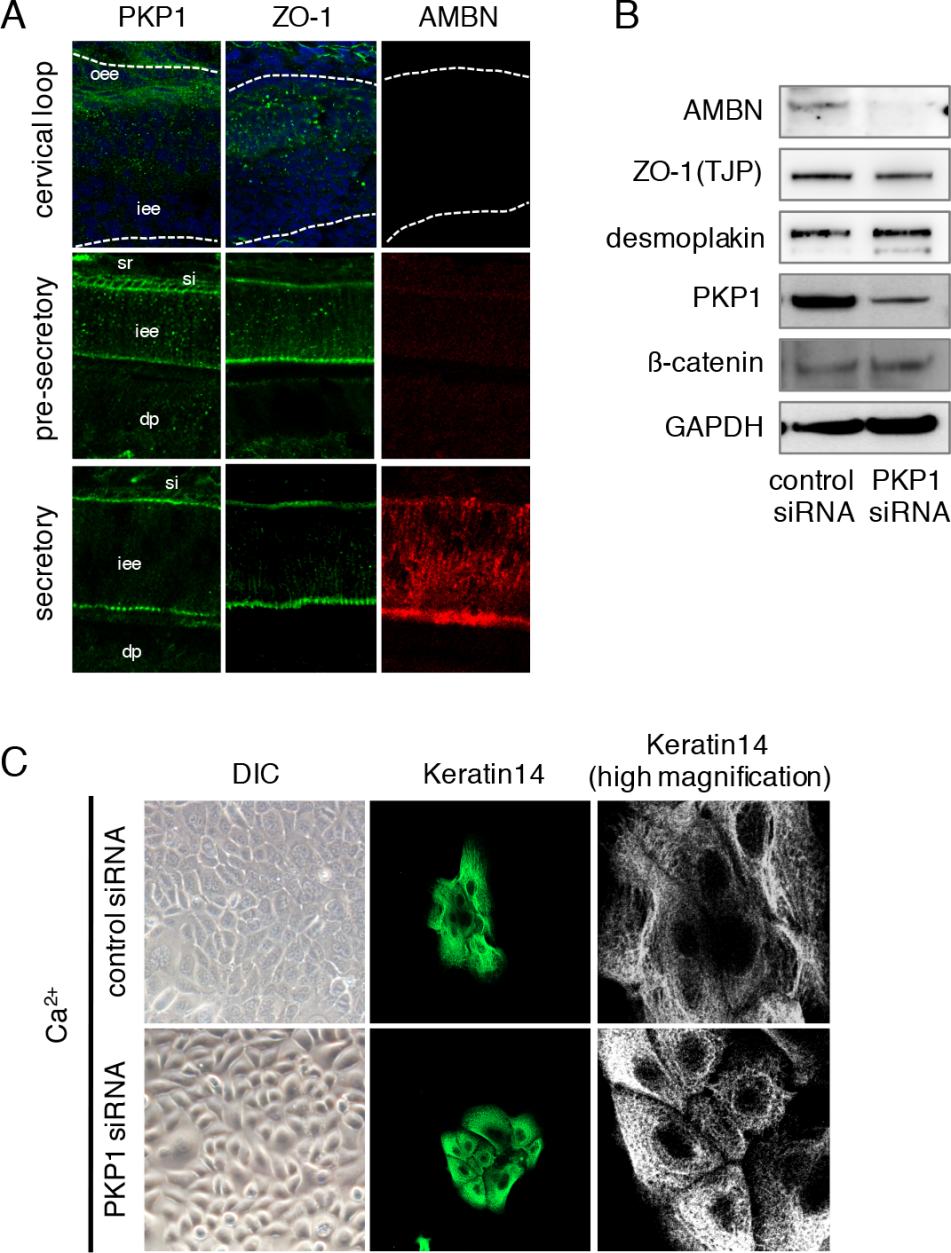
Expression patterns of PKP1 and ZO-1 in P1 incisors. A, PKP1 (green), ZO-1 (green), and AMBN (red) expression in cervical loop of P1 incisors at pre-secretory and secretory stages, as determined by immunocytochemistry. Nuclei were stained with DAPI (blue). iee, inner enamel epithelium; oee, outer enamel epithelium; sr, stellate reticulum; si, stratum intermedium; dp, dental papilla. B, CLDE cells were cultured after transfection with control or *Pkp1* siRNA in the presence of Ca^2+^ for 48 h, then the expressions of AMBN, ZO-1, Desmoplakin, PKP1, and β-catenin were assessed by western blotting. GAPDH was used as a loading control. C, CLDE cells were cultured after transfection with control or *Pkp1* siRNA in the presence of Ca^2+^ for 48 h. Differential interference contrast image (left) and immunolabeling of keratin 14 (green; center panel and enlarged in right panel) are shown.

Wnt signaling plays an important role in the early stages of tooth development and our results implicate PKP1 in this process. Inner dental epithelial cells differentiate into ameloblasts that secrete enamel matrix proteins. At this stage, ameloblasts alter their shape to a columnar form as a result of cell-cell communication. To examine the dynamics of PKP1 translocation during ameloblast differentiation in vivo, we evaluated PKP1, ZO-1, and AMBN expressions in P1 mouse incisors using immunohistochemistry. Incisors grow continuously throughout the life of the mouse owing to dental epithelial stem cells residing in the cervical loop. It is therefore possible to observe all stages of ameloblast differentiation in tissue sections, including the pre-secretory and secretory stages (Fig 6A). At P1 during early differentiation, PKP1 was localized in the cytosol near the cervical loop. During the pre-secretory and secretory stages, it translocated to the plasma membrane, and was present between ameloblasts and the stratum intermedium or enamel matrix. Punctate PKP1 distribution was also observed in ameloblast-ameloblast junctions (Fig 6A). PKP1 is known to be present in desmosomes and involved in cell-cell adhesion, thus we examined the localization of ZO-1 in order to determine the involvement of tight junction complexes in tooth development. Interestingly, the expression pattern of ZO-1 in incisors was similar to that of PKP1 during the pre-secretory and secretory stages (Fig 6A), suggesting that PKP1 may associate with tight junctions during tooth development.

To verify our findings, we performed knockdown of *Pkp1* in CLDE cells and examined the effect on ameloblast differentiation. Loss of *Pkp1* decreased AMBN, but not ZO-1 or desomoplakin levels, as determined by western blotting findings (Fig 6B), indicating that PKP1 is critical for ameloblast differentiation. *Pkp1* siRNA treatment also abrogated the alteration in cell shape induced by Ca^2+^, yielding cells that were round (Fig 6C). In addition, *Pkp1* knockdown induced formation of a gap in cell-cell junctions, as visualized by immunolabeling of keratin 14 (Fig 6C). These results indicate that PKP1 is involved in ameloblast differentiation via an association with cell-cell adhesion complexes.

### PKP1 regulates ZO-1 localization via cell adhesion machinery

**Fig 7:**
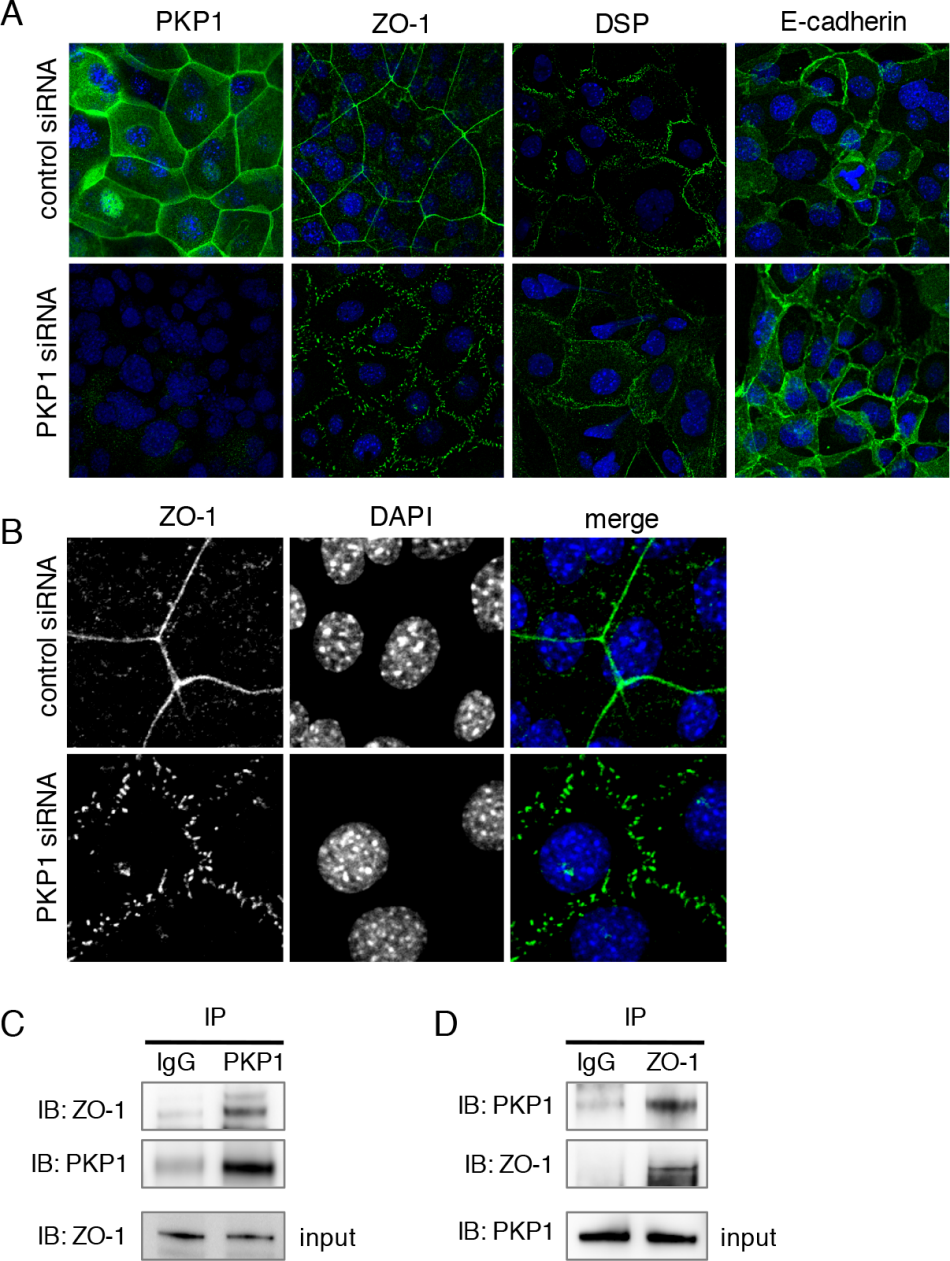
PKP1 regulates ameloblast differentiation and ZO-1 localization. A, PKP1, ZO-1, Desmoplakin, and E-cadherin expressions in CLDE cells transfected with control or *Pkp1* siRNA, as detected by immunocytochemistry. Nuclei were counterstained with DAPI. B, Enlarged image of disturbed ZO-1 expression following *Pkp1* knockdown. Nuclei were counterstained with DAPI. C, CLDE cells were cultured for 1 week in the presence of Ca^2+^. Immunoprecipitation was performed with control IgG and anti-PKP1 antibodies, then ZO-1 and PKP1 binding was assessed by western blotting. Input lysates were confirmed by probing for ZO-1 (bottom). D, Immunoprecipitation was performed with control IgG and anti-ZO-1 antibodies, then PKP1 and ZO-1 binding was assessed by western blotting. Input lysates were confirmed by probing for PKP1 (bottom).

To clarify the role of cell adhesion proteins in regulation of tooth development by PKP1, we examined the localization of the cell adhesion molecules ZO-1 (tight junctions), Desomoplakin (desmosomes), and E-cadherin (adherens junctions) by immunohistochemistry. Interestingly, the expression of ZO-1, but not of Desomoplakin or E-cadherin, in the plasma membrane was disrupted and appeared punctate upon *Pkp1* knockdown (Fig 7A and 7B), suggesting that PKP1 regulates ZO-1 distribution at the plasma membrane of dental epithelial cells. Cell adhesion molecules are involved in ameloblast differentiation, stabilization, and polarity, and our findings indicate that PKP1 controls ZO-1 distribution and may regulate ameloblast differentiation. We then examined whether PKP1 and ZO-1 directly interact by immunoprecipitation. In the presence of Ca^2+^, PKP1 was precipitated from CLDE cell lysates using an anti-ZO-1 antibody and vice versa (Fig 7C and 7D). These results indicate that PKP1 physically interacts with and controls the distribution of ZO-1 at the plasma membrane of dental epithelial cells, and may thereby regulate cell-cell adhesion and ameloblast differentiation.

## Discussion

The present study showed that PKP1 is important for tooth development and regulates ZO-1 distribution in the dental epithelium. It was previously reported that a mutation in the *Pkp1* gene results in ectodermal dysplasia/skin fragility syndrome [14]. In affected patients, desmosomes in the skin are small and poorly formed, with wider spaces between keratinocytes and perturbed desmosome/keratin interactions, implying an important role for PKP1 in both cutaneous cell-cell adhesion and epidermal morphogenesis. Ablation of the *Pkp2* gene in mice resulted in lethal alterations in heart morphogenesis and stability at mid-gestation [26] caused by dissociation of the cytoskeletal linker protein Desmoplakin desmoplakin from adherens junction plaques that connect granular aggregates in the cytoplasm of cardiomyocytes, suggesting that PKP2 is necessary for assembly of junctional complexes, as well as an essential morphogenic factor and architectural component of the developing heart. The differences in phenotypes between *Pkp1* and *Pkp2* mutants may be explained by tissue-specific expression of these genes. In the present experiments, *Pkp1* was found to be specifically expressed in teeth and skin.

PKP1 contains an armadillo repeat domain at the N terminus. Armadillo repeat proteins mediate protein-protein interactions in diverse cellular processes, including cell junction assembly, nuclear transport, and transcriptional activation. Members of this family, which include the adherens junction component and transcription factor β-catenin, and nuclear import protein importin-α, contain multiple copies of the 42-a.a. armadillo repeat motif [25]. The N terminus of PKP1 has been shown to interact with the desmosome proteins Desmoglein 1, Desmoplakin, and Keratin [27]. We found that N-terminal deletion of PKP1 abolished its Wnt-activated nuclear translocation, indicating that this region may be essential for its nuclear translocation and activity, the same as for β-catenin. Indeed, PKP1 was mainly detected in nuclei in our study. Moreover, chimeric proteins containing the PKP1 N terminus and PKP3 armadillo repeat domain were localized in nuclei of dermal cells, whereas those containing the PKP3 N terminus and PKP1 armadillo repeat domain were localized in desmosomes and cytosol [28]. PKP is also known to regulate mRNA expression, desmosomal cadherin trafficking, and insulin-induced cell proliferation [29–32], thus it directly interacts with single-stranded DNA and Ets family transcription factors [28, 33], suggesting that it regulates proliferation through control of gene expression in the nucleus in a manner similar to β-catenin.

Wnt signaling is crucial for maintaining the balance between proliferation and differentiation throughout embryogenesis and tissue regeneration. Loss of Wnt3a during development resulted in truncation along the A-P axis, with loss of caudal somites and tail bud development. Embryos in which Wnt5a has been disrupted show similar axial truncation, along with incomplete growth of distal limbs, impaired lung morphology, and pituitary gland abnormalities [34]. In addition, transgenic mice overexpressing the Wnt inhibitor Dickkopf 1 showed reduced expression of bone morphogenetic protein (Bmp)2, Bmp4, Gli1, Lef1, Ectodysplasin A receptor, Keratin17, and β-catenin, as well as aberrations in hair, tooth, and limb formation [35]. These results indicate that Wnt-β-catenin signaling is important for organogenesis and especially for development of ectodermal organs, including teeth. In the present experiments, treatment with Wnt3a, as well as overexpression of full-length but not N-terminal-deleted PKP1, induced dental epithelial cell proliferation and TCF/LEF activation, while those effects were abolished by siRNA-mediated knockdown of *Pkp1*, indicating that PKP1 may be a downstream effector of Wnt signaling that regulates dental epithelial cell proliferation. β-catenin is also known to be important for tooth germ development, as expression of a constitutively active form and tissue-specific deletion of the negative Wnt regulator Adenomatous polyposis coli results in formation of supernumerary teeth [5, 36, 37]. However, the differences between the functions of PKP1 and β-catenin in tooth development are unclear, and should be addressed in future studies. Our preliminary experiments showed that Pkp-1 is expressed in dental epithelium, but not dental mesenchyme. In contrast, β-catenin was found to be strongly expressed in mesenchyme outside of the tooth germ during the embryonic stage and then decreased during the postnatal stage, suggesting that Pkp-1 but not β-catenin is important for dental epithelium differentiation.

Epithelial cell polarization and cell-cell communication are important for tooth development. Our previous study showed that mutant mice lacking the basement membrane molecule Laminin alpha 5 had small teeth and lacked cell polarity in the dental epithelium [20]. Furthermore, deficiency in the enamel matrix protein AMBN resulted in loss of polarization and accelerated the proliferation of ameloblasts, leading to amelogenesis imperfecta and epithelial odontogenic tumor formation [38]. Communication through gap junctions also regulates ameloblast differentiation and bone formation, as a mutation in the gene encoding Connexin 43 has been reported to result in development of oculodentodigital dysplasia, a predominantly autosomal disease characterized by syndactyle, microdontia, enamel hypoplasia, and craniofacial abnormalities [39, 40]. These results suggest that cell-matrix and cell-cell interactions are necessary for dental epithelium proliferation and differentiation. ZO-1, originally reported as a component of tight junctions [41], is a member of the membrane-associated guanylate kinase family of proteins that function in protein targeting, signal transduction, and determination of cell polarity [42]. ZO proteins bind to other junctional transmembrane proteins such as Occludin and Claudin, as well as to F-actin and other regulatory components of the cytoskeleton [43, 44]. Mutations in ZO-1 alter gap junction size and organization [45], and ZO-1 deficiency has revealed its involvement in control of cell shape via regulation of the cytoskeleton [46]. Moreover, ZO-1 knockout embryos die at mid-gestation [47] and podocyte-specific deletion of ZO-1 leads to glomerulosclerosis, suggesting that it regulates the structure and function of podocyte filtration slits [48]. However, the role of ZO-1 in tooth development has not been clearly demonstrated. In the present study, we found that PKP1 and ZO-1 were similarly expressed during dental epithelium differentiation. Furthermore, dental epithelium with *Pkp1* knockdown showed disrupted ZO-1 organization and these 2 proteins have been shown to directly interact by immunoprecipitation. Together, these results suggest that PKP1 regulates ZO-1 localization and, accordingly, ameloblast differentiation during tooth development.

This is the first report describing the expression and role of PKP1 in tooth development. Our findings provide insight into the basic function of PKP1 and suggest possible applications in tooth germ regeneration, as well as show it to be a potential target in treatment of diseases such as ectodermal dysplasia/skin fragility syndrome.

## Supporting Information

S1 Fig Expression pattern of CTNNB1 in developing teeth. A, qRT-PCR analysis of *Ctnnb1* expression in teeth, skin, lungs, livers, kidneys, hearts, eyes, and brains of E14.5 embryos after normalization to *Gapdh* mRNA expression. B, qRT-PCR analysis of *Ctnnb1* expression in teeth obtained from E11 to P7 after normalization to *Gapdh* mRNA expression. C, CTNNB1 (green) expression in E13, E14, E16, and P1 mice, as detected by immunocytochemistry. Broken lines represent the basement membrane of teeth. Enlarged images are shown below each panel. de, dental epithelium; dm, dental mesenchyme; iee, inner enamel epithelium; sr, stellate reticulum; si, stratum intermedium; dp, dental papilla.
(TIF)

S2 Fig Wnt3a induces translocation of β-catenin and endogenous PKP1 from membrane to nucleus. A, B, CLDE cells were cultured with or without Wnt3a for 24 h. Nuclear translocation of endogenous PKP1 and β-catenin was detected by immunohistochemistry. Nuclei were stained with DAPI. C, CLDE cells were transfected with β-catenin-EmGFP, then treated with or without Wnt3a for 24 h. Nuclear translocation was detected by confocal microscopy. Nuclei were stained with DAPI.
(TIF)
