# Supplementary figures and images for: Plakophilin-1, a Novel Wnt Signaling Regulator, Is Critical for Tooth Development and Ameloblast Differentiation
Source: PLoS One. 2016 Mar 24;11(3):e0152206. doi: 10.1371/journal.pone.0152206 (PMC4806907; doi:10.1371/journal.pone.0152206)

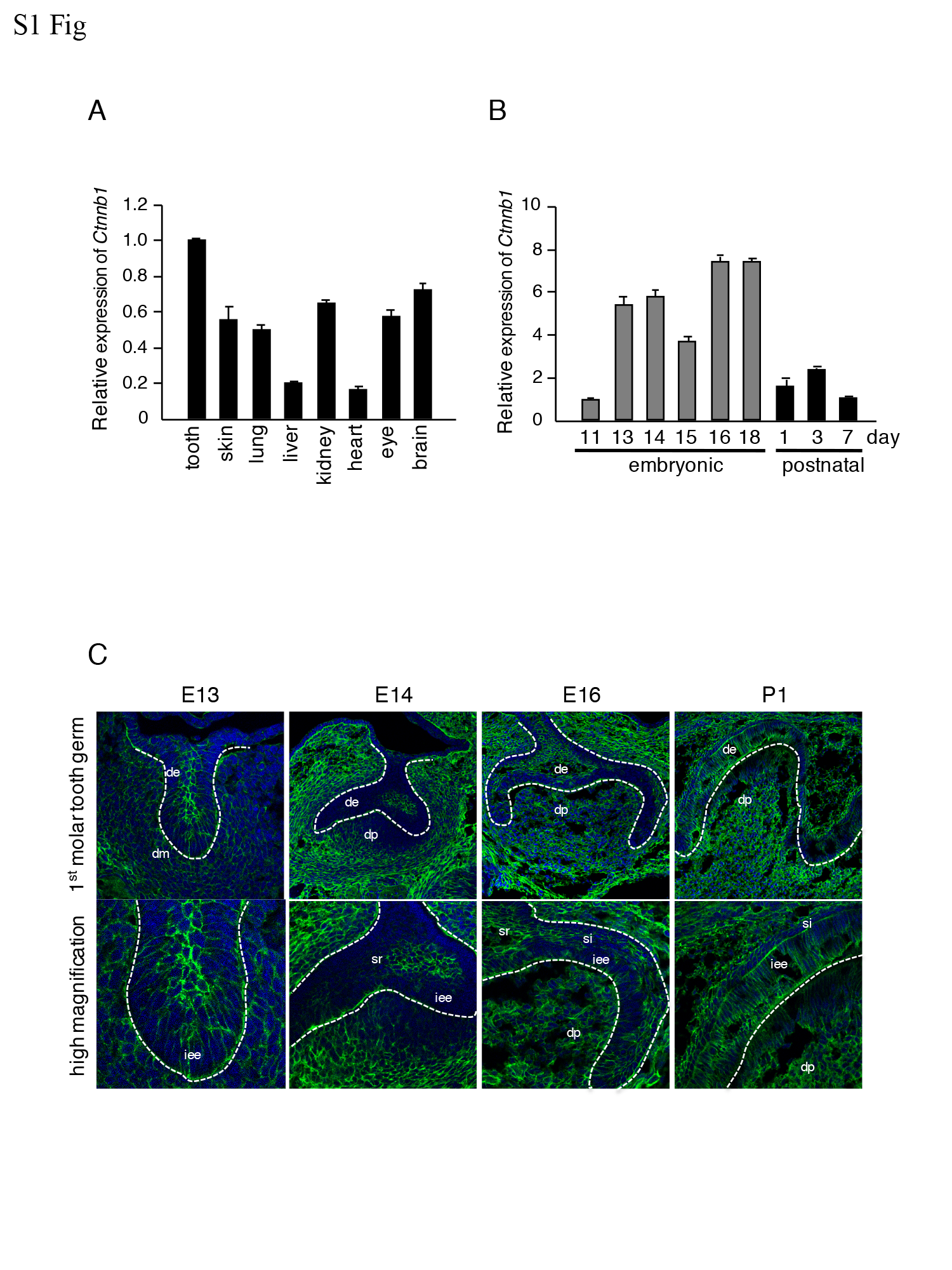

Supplement: S1 Fig — A, qRT-PCR analysis of Ctnnb1 expression in teeth, skin, lungs, livers, kidneys, hearts, eyes, and brains of E14.5 embryos after normalization to Gapdh mRNA expression. B, qRT-PCR analysis of Ctnnb1 expression in teeth obtained from E11 to P7 after normalization to Gapdh mRNA expression. C, CTNNB1 (green) expression in E13, E14, E16, and P1 mice, as detected by immunocytochemistry. Broken lines represent the basement membrane of teeth. Enlarged images are shown below each panel. de, dental epithelium; dm, dental mesenchyme; iee, inner enamel epithelium; sr, stellate reticulum; si, stratum intermedium; dp, dental papilla. (TIF) [file pone.0152206.s001.tif]

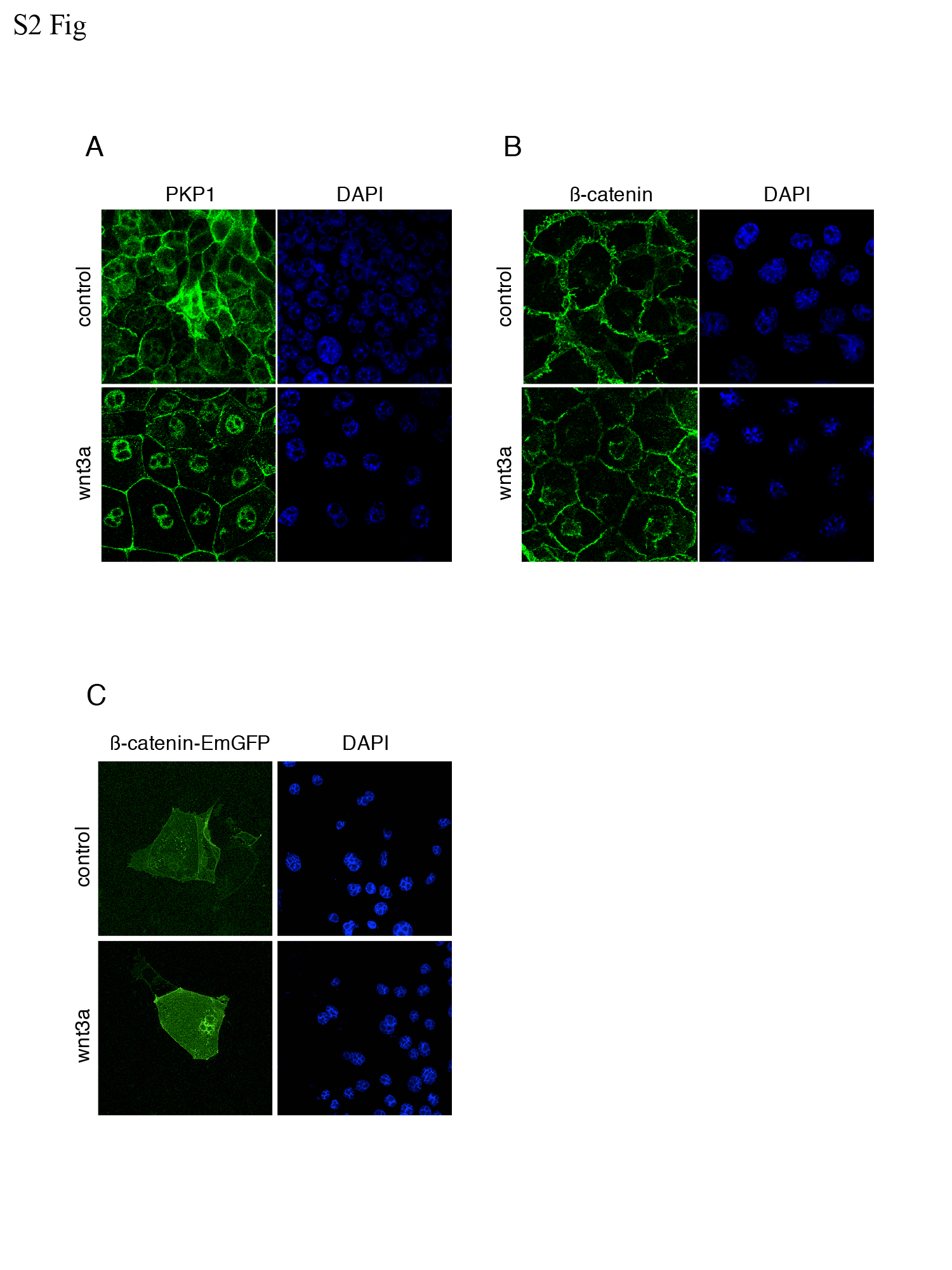

Supplement: S2 Fig — A, B, CLDE cells were cultured with or without Wnt3a for 24 h. Nuclear translocation of endogenous PKP1 and β-catenin was detected by immunohistochemistry. Nuclei were stained with DAPI. C, CLDE cells were transfected with β-catenin-EmGFP, then treated with or without Wnt3a for 24 h. Nuclear translocation was detected by confocal microscopy. Nuclei were stained with DAPI. (TIF) [file pone.0152206.s002.tif]
